# A new therapeutic proposal for writer’s cramp: a case report

**DOI:** 10.1590/S1516-31802010000200010

**Published:** 2010-03-04

**Authors:** Flavia Quadros Boisson Waissman, João Santos Pereira, Osvaldo José Moreira Nascimento

**Affiliations:** 1 MSc. Physiotherapist, Department of Neurology, Universidade Federal Fluminense (UFF), Niterói, Rio de Janeiro, Brazil.; II MD, PhD. Adjunct professor of Neurology, Head of Movement Disorders Section, Department of Neurology, Pedro Ernesto University Hospital, Universidade do Estado do Rio de Janeiro (UERJ), Rio de Janeiro, Brazil.; III MD, PhD. Titular professor of Neurology, Department of Neurology, Universidade Federal Fluminense (UFF), Niterói, Rio de Janeiro, Brazil.

**Keywords:** Writer’s cramp, Focal dystonia, Dystonic disorders, Movement disorder, Handwriting, Câimbra do escrivão, Distonia focal, Distúrbios distônicos, Distúrbios do movimento, Escrita manual

## Abstract

**CONTEXT:**

Writer’s cramp is a kind of focal hand dystonia that appears when individuals are writing. Since pharmacological treatment has not shown the desired therapeutic response, a study on immobilization of the damaged musculature was performed on two individuals with writer’s cramp, using splints with the objective of reducing the handwriting abnormalities.

**CASE REPORT:**

Two patients presenting writer’s cramp who had previously undergone different therapies, including botulinum toxin, without an adequate response, participated in a body awareness program, followed by immobilization of the hand musculature damaged by dystonia, by means of splints, with handwriting training. At the end of the procedure, objective and subjective improvements in the motor pattern of writing could be observed. The immobilization of the dystonic musculature of the hand by means of splints and the motor training of handwriting helped to improve and consequently to reduce the dystonic component observed in the writer’s cramp.

## RESUMO

**CONTEXTO:**

A câimbra do escrivão é um tipo de distonia focal da mão que aparece quando a pessoa escreve. Como o tratamento farmacológico não tem mostrado a resposta terapêutica almejada, um estudo sobre a imobilização da musculatura comprometida foi feito em dois indivíduos com câimbra do escrivão, utilizando órteses com o objetivo de reduzir as alterações da escrita.

**RELATO DE CASO:**

Dois pacientes apresentando câimbra do escrivão que haviam sido submetidos previamente a terapêuticas, inclusive toxina botulínica, sem resposta adequada, participaram de um programa de consciência corporal, seguido de imobilização por órteses da musculatura da mão comprometida pela distonia, com treinamento da escrita. Ao término, evidenciaram melhora objetiva e subjetiva no padrão motor da escrita. A imobilização por órteses da musculatura distônica da mão e o treinamento motor da escrita favorecem a melhora e, consequentemente, a redução do componente distônico observado na câimbra do escrivão.

## INTRODUCTION

Writer’s cramp is shown by involuntary muscle contractions in the arm or hand while writing. Compression, paper destruction, pain and difficulty in holding a pen may occur. The abnormality of sensory-motor integration may be due to impairment of afferent activity and a sensory-motor training program can modify this disorder.^1^

The effects of the use of splints in the cases of two patients with writer’s cramp were investigated.

## CASE REPORT

Two patients with diagnoses of writer’s cramp (one male and one female) were accepted for evaluation. The institution’s Ethics Committee approved this study and the patients signed a free and informed consent statement.

The first patient, a right-handed 24-year-old white male, had presented difficulty in writing for six years, followed by intense pain in the thenar region, seconds after beginning the task. The second patient, a right-handed 44-year-old white female, had presented difficulty in writing for 28 years, followed by pain and fatigue in her forearm and hand, plus extension and radial deviation of the wrist.

Functional motor evaluation was performed on the hand using the Burke-Fahn-Marsden scale (BFMS) and Jedynak’s protocol, and an analog pain scale.^2^ The rehabilitation training was administered over an eight-week period, in two phases of four weeks each, twice a week for 60 minutes each session. During the first phase, body awareness training using relaxation techniques was administered. During the second phase, appropriate splints were used on the affected musculature of the fingers, in order to inhibit dystonic action. Similar exercises at home were scheduled for 30 minutes a day.

For the first patient, the functional motor examination showed dystonic postures of the hand and forearm during writing, with index finger and thumb extension, wrist flexion and excessive pressure used to hold the pen. The Jedynak score for writing quality was two. Using the BFMS, the scores for the dystonic effect and for handwriting were both two. A few seconds after beginning to write, a pain in the fingers and thenar region appeared, which were scored as three and ten, respectively.

**Figure 1.**
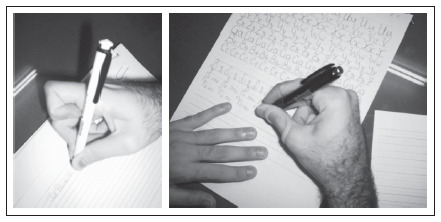
Dystonic posture of forefinger before the sensory-motor training (left) and the improvement following the program (right). This posture was maintained without the splint.

After the rehabilitation, dystonic posture and excessive pressure were no longer found, thus showing better motor control. Although there was an improvement, the handwriting still presented occasional irregularities, and was scored as one, on the Jedynak protocol. The score for the functional motor characteristics evaluated by BFMS was zero and for writing disability, one (**Figure 1**). The pain in the fingers and thenar region while writing were scored as one and eight, respectively.

The second patient showed forearm pronation, flexion and radial deviation of the right wrist, flexion with overlapping of the index finger in relation to the thumb, and excessive grip on the pen and pressure on the paper. The pain in the fingers and thenar region was scored as three and ten, respectively. The writing quality was three. From the BFMS, the score for dystonic movement of the arm region was six and for writing disability, two.

**Figure 2.**
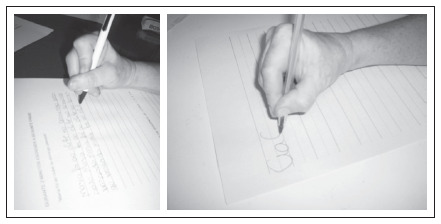
Dystonic posture of patient 2 before (left) and after the sensory-motor training, showing an improvement in motor control when holding a pen (right).

The forearm pronation and dystonic fingers showed great improvement. The gripping of the pen and pressure on the paper were greatly decreased. The quality of handwriting was scored as one using the Jedynak protocol. From the BFMS, the score for dystonic movement was four and for writing disability, one (**Figure 2**). The pain in the fingers and thenar region was scored as zero and nine, respectively. Subjectively, there was a 30% to 40% improvement in the writing process.

## DISCUSSION

In our study, the body awareness training had the result that compensatory maneuvers no longer occurred after the training period. These observations resemble the results from body reeducation among musicians with focal hand dystonia.^3^

In both patients studied, reductions in pain and improvements in handwriting quality, character formation and pressure on the paper occurred, comparing the situations before and after the program.

These patients with writer’s cramp maintained the 30 to 50% improvement in their symptoms after the removal of the immobilization splints. Another study found that 50% of the patients maintained a marked improvement.^4^ Our findings, although preliminary, showed slight to moderate improvement.

Although another report described worsening of writing after daily practice was discontinued, the benefits acquired during this training with splints can be maintained for six months.^4^ Studies using a larger sample would be necessary, although these results are not far from those observed with the use of botulinum toxin, which produced satisfactory results in 50% to 70% of the cases and only 16.4% of those patients used oral medication.^5^

Considering the results from our investigation, and because of the major difficulty in diagnosing and providing therapy for this neurological disorder, we carried out a systematic analysis of the indexed articles published since 1998, in order to provide a better foundation for our study. We searched using the term "writer’s cramp" in the Lilacs (Literatura Latino-Americana e do Caribe em Ciências da Saúde), Embase (Excerpta Medica Database), Medline and Cochrane Library databases, using DeCS (Descritores em Ciências da Saúde) and MeSH (Medical Subject Headings). Of the 715 references found in Medline alone, 115 really referred to writer’s cramp or focal hand dystonia in their titles. In Lilacs, out of seven papers, only one paper related to the subject^6^ (**Table 1**).

**Table 1.** Total numbers of papers indexed by Lilacs (Literatura Latino-Americana e do Caribe em Ciências da Saúde), Embase (Excerpta Medica), Medline and the Cochrane Library, with regard to the term "writer’s cramp" (1998 to 2008)

Data	Search strategy	Results
Medline	Writer’s cramp (MeSH)	10 reviews
		2 experiments
		4 randomized controlled trials
		27 case reports
		8 editorials/communications
		64 clinical trials
Lilacs	Writer’s cramp (MeSH)	1 case report

Medline = Medical Literature Analysis and Retrieval System Online; MeSH = Medical Subject Headings.

Among these papers, there were six studies^7-12^ using botulinum toxin A, with different results. In relation to splint immobilization, the same number of studies was found^1,4,13-16^ and two of these were prospective studies^1,13^ Most of the studies related to diagnostic methods or functional tests. It could be seen that this rehabilitative procedure is very rarely used. This may be because it is unknown to some health professionals, or even because of difficulty in adherence to treatment among the patients.

## CONCLUSION

The therapeutic response to noninvasive treatment for writer’s cramp is efficient with regard to writing quality, morphofunctional characteristics and manual ability.
